# Identification of a Transcriptomic Network Underlying the Wrinkly and Smooth Phenotypes of Vibrio fischeri

**DOI:** 10.1128/JB.00259-20

**Published:** 2021-01-11

**Authors:** Alba Chavez-Dozal, William Soto, Michele K. Nishiguchi

**Affiliations:** a Department of Biology, New Mexico State University, Las Cruces, New Mexico, USA; b Department of Human Factors and Behavioral Neurobiology, Embry-Riddle Aeronautical University, Daytona Beach, Florida, USA; c Department of Biology, College of William and Mary, Williamsburg, Virginia, USA; d Department of Molecular and Cell Biology, School of Natural Sciences, University of California, Merced, Merced, California, USA; Université de Montréal

**Keywords:** rugose (wrinkly)/smooth phenotype, *Vibrio fischeri*, transcriptome, biofilm, *Vibrio*, phenotype, smooth, wrinkly

## Abstract

Vibrio fischeri is a cosmopolitan marine bacterium that oftentimes displays different colony morphologies, switching from a smooth to a wrinkly phenotype in order to adapt to changes in the environment. This wrinkly phenotype has also been associated with increased biofilm formation, an essential characteristic for V. fischeri to adhere to substrates, to suspended debris, and within the light organs of sepiolid squids. Elevated levels of biofilm formation are correlated with increased microbial survival of exposure to environmental stressors and the ability to expand niche breadth. Since V. fischeri has a biphasic life history strategy between its free-living and symbiotic states, we were interested in whether the wrinkly morphotype demonstrated differences in its expression profile in comparison to the naturally occurring and more common smooth variant. We show that genes involved in major biochemical cascades, including those involved in protein sorting, oxidative stress, and membrane transport, play a role in the wrinkly phenotype. Interestingly, only a few unique genes are specifically involved in macromolecule biosynthesis in the wrinkly phenotype, which underlies the importance of other pathways utilized for adaptation under the conditions in which *Vibrio* bacteria are producing this change in phenotype. These results provide the first comprehensive analysis of the complex form of genetic activation that underlies the diversity in morphologies of V. fischeri when switching between two different colony morphotypes, each representing a unique biofilm ecotype.

**IMPORTANCE** The wrinkly bacterial colony phenotype has been associated with increased squid host colonization in V. fischeri. The significance of our research is in identifying the genetic mechanisms that are responsible for heightened biofilm formation in V. fischeri. This report also advances our understanding of gene regulation in V. fischeri and brings to the forefront a number of previously overlooked genetic networks. Several loci that were identified in this study were not previously known to be associated with biofilm formation in V. fischeri.

## Abstract (precis)

The wrinkly bacterial colony phenotype has been associated with increased squid host colonization in V. fischeri. The significance of our research is in identifying the genetic mechanisms that are responsible for heightened biofilm formation in V. fischeri.

## INTRODUCTION

Vibrio fischeri is a bioluminescent bacterium that establishes a mutualistic association with sepiolid squids and monocentrid fishes (1, 2). The mutualism is established when the host provides an appropriate niche for the bacteria to reproduce at much higher rates and the bacteria provide bioluminescence in the form of counterillumination that is used by the squid to avoid predation (2). During colonization, V. fischeri is capable of forming biofilms both inside the host (during symbiosis) and in the environment (during its free-living state) (3). Newly hatched *Euprymna* squid are bacterium free (or aposymbiotic) inside the light organ (LO) complex. However, there is rapid formation of a biofilm layer inside the host LO within hours of infection, where this bacterial population can be observed in the ciliated fields of the squid by confocal and electron microscopy (3).

Previous studies have revealed that two distinct host-independent bacterial phenotypes are related to the ability to survive and form biofilms in seawater and inside the squid host LO that provide survival and fitness advantages in those particular environments (2, 4–6). The phenotypes that have been observed are variable but can be summarized into smooth and wrinkly (or rugose) colonies. Therefore, V. fischeri can alter its phenotype and reversibly switch from a smooth colony morphology to a wrinkly colony morphology characterized by increased production of extracellular polysaccharide (EPS) and biofilm formation (7).

**FIG 1:**
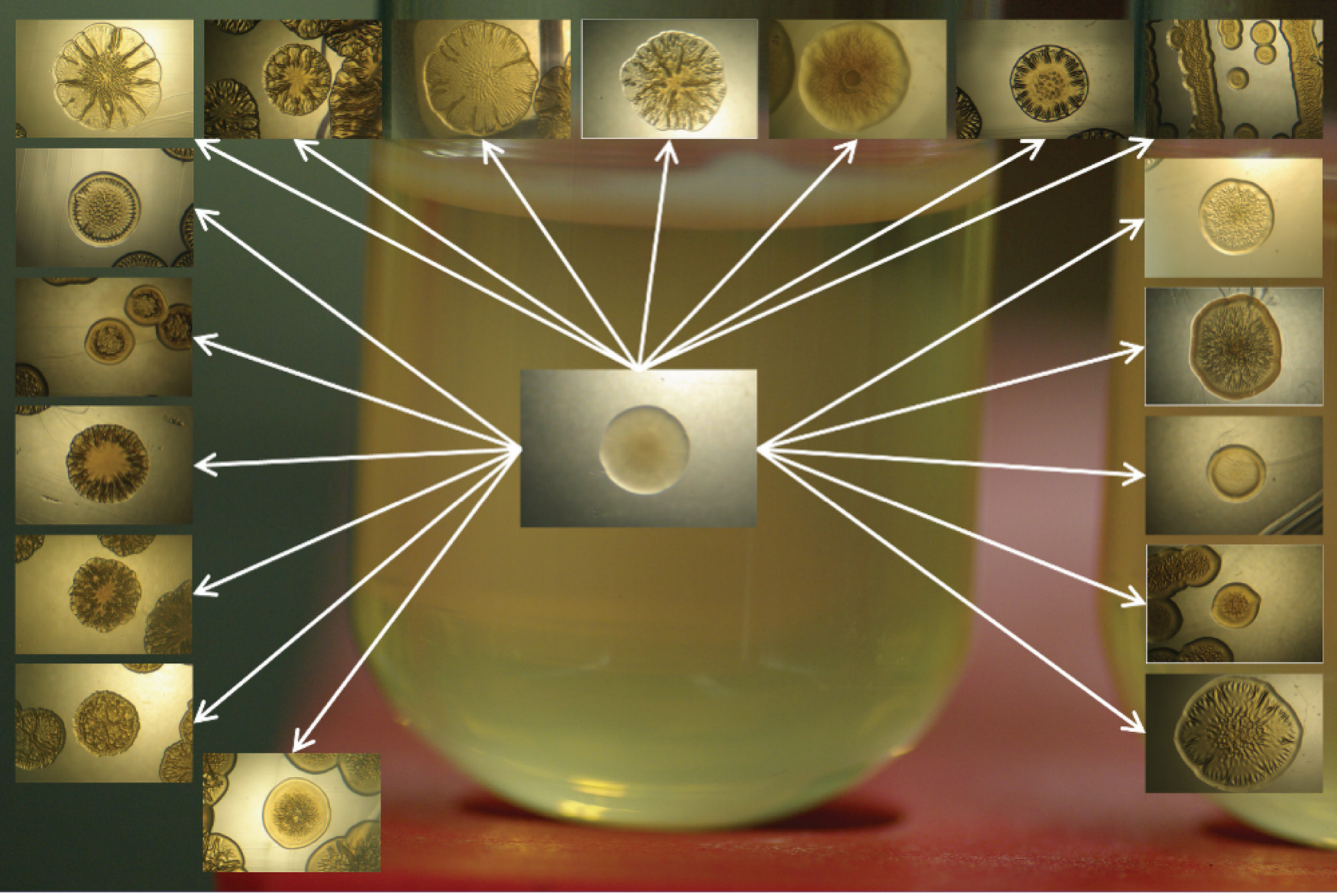
Adaptive phenotypes in liquid static microcosms give rise to the wrinkly spreader. Shown are different colonies isolated from the culture grown in modified seawater-tryptone (MSWT; 1.0% [wt/vol] tryptone, 0.5% [wt/vol] yeast extract, 0.3% [wt/vol] glycerol, 513.3 mM NaCl, 50.0 mM MgSO_4_, 10.0 mM CaCl_2_, 10.0 mM KCl, 0.01 mM FeSO_4_, 10.0 mM NH_4_Cl, 0.33 mM K_2_HPO_4_, and 50.0 mM Tris [pH 7.5]). Transferred to agar plates, and incubated for 3 days at 28°C. Wrinkly spreader colonies have irregular, multilobed circumferences and a flattened and wrinkled surface.

Colony morphology variations affect host colonization quite differently, demonstrating that the rugose or wrinkly variant provides a colonization advantage over the smooth phenotype (7, 8). Adaptive radiation is a key element in the development of this microecological diversity (5). Interestingly, one study previously demonstrated that V. fischeri wrinkly populations were derived from smooth ancestors after a simulated microbial evolution experiment in selected microcosms (Fig. 1) (5). The derived wrinkly colonies showed an increased ability to produce EPS and to form biofilms (5). Additionally, wrinkly colonies demonstrated a clear advantage in host colonization in squid hosts in competition studies performed using both colony phenotypes of V. fischeri (5, 8).

Previous studies have highlighted the phenotypical differences between the two colony phenotypes in bacteria, including *Pseudomonas*, where the wrinkly phenotypes evolve repeatedly and show interesting fitness differences from the smooth counterpart (9). Additionally, multiple *Vibrio* species, including V. cholerae, V. harveyi, and, more recently, V. fischeri, have been examined for this change in phenotype (10–15); however, little is known about the genetic underpinnings of these differences. Only a few studies have examined the role of transcriptional activation of the *vps* (*Vibrio* polysaccharide) gene cluster in wrinkly colonies (3, 16). The *vps* cluster leads to production of (i) transcriptional activators of multiple wrinkly phenotype-associated operons (including genes responsible for increasing biofilm formation), (ii) multiple kinase proteins linked to the wrinkly phenotype by some unknown mechanism, (iii) genes responsible for luminescence, quorum sensing (QS), or bacterial communication, and (iv) activation of the second messenger c-di-GMP which also induces production of EPS and biofilm formation and regulates flagellar biosynthesis and twitching motility (3). Those studies provided clear evidence of transcriptional activation of genes that are crucial for colony phenotype, and yet there is still much about the molecular basis of the wrinkly phenotype that is not understood, especially in V. fischeri.

In the present study, we implemented a differential transcriptional analysis of smooth and wrinkly colonies in order to identify genes involved in the molecular switch between these two phenotypes. We selected three V. fischeri strains, including one isolated from the squid host Euprymna scolopes (ES114), one isolated from the monocentrid fish host Cleidopus gloriamaris (CG101), and one free-living strain isolated from seawater (ATCC 7744). All these strains have been previously studied and show evolutionary adaptation from the smooth to the wrinkly phenotype (5). Along with genes involved in metabolism, cell structure, and membrane transport, our study identified genes associated with stress responses in the wrinkly phenotype. These interesting results demonstrate how microorganisms such as V. fischeri can utilize diverse mechanisms to evolve alternative biofilms for their biphasic life history strategy.

## RESULTS AND DISCUSSION

In previous studies, it has been observed that bacterial adaptation often leads to establishment of significantly altered phenotypes. For example, when V. fischeri is incubated *in vitro* in different microcosms, it displays a wide range of phenotypes (4, 5, 7). Of these, the colonization of the air-liquid interface by the wrinkly phenotype is the most spectacular (Fig. 1). Recent experimental evolution studies performed by Soto et al. (5) demonstrated that the wrinkly spreader overcomes the smooth morph within days of squid host infection, having a significant fitness advantage over the ancestral strain. In addition, examination of the wrinkly spreaders has provided a mechanistic explanation linking phenotype with fitness improvement through quantitative differences in biofilm formation, motility, and carbon source utilization (7). The mechanistic explanation of the wrinkly spreader success is an exemplar of evolutionary adaptation, linking molecular biology with evolutionary biology and host colonization, as well as providing insight into the symbiont’s ability to adapt to multiple environments. Previous studies in both *Pseudomonas* and *Escherichia* have linked the wrinkly spreader phenotype to multiple genes involved in lipopolysaccharide production, biofilm formation, and the production of second messenger c-di-GMP (14, 17). Given that V. fischeri has a more in-depth life history strategy that balances host colonization with environmental survival, the data provide a unique outlook on how multiple genes for biofilm production are coopted for both life styles.

In the present study, we evaluated differences in gene expression of V. fischeri from two different colony phenotypes, smooth and wrinkly. We performed transcriptome sequencing (RNA-Seq)-based analysis to explore the genome-wide response that led to each phenotype. For this purpose, a total of 6 Illumina libraries were sequenced as approximately 50-bp reads. The Illumina reads correspond to a representative sample of different clones of each V. fischeri strain from different inoculum sources, including two symbiotic (ES114 and CG101) and one free-living (V. fischeri ATCC 7744). Symbiotic strain ES114 is the V. fischeri light organ symbiont obtained from the bobtail squid *Euprymna scolopes* that has been fully sequenced and used in different research studies for over 20 years. In contrast, V. fischeri symbiotic strain CG101 was isolated from the monocentrid fish host *Cleidopus gloriamaris* and has been studied but in less detail than the squid strains. Finally, strain V. fischeri ATCC 7744 is a free-living commercially available strain which has also been used as a reference strain for genetic analysis studies since 1993. These three strains were selected due to the unique nature of their source (isolated either from two different hosts or from a free-living environment) in an effort to study the effects of natural strain variations on genetic regulation of the wrinkly and smooth phenotypes.

On average, the libraries had approximately 15,000,000 million reads, of which 80% were uniquely mapped to the V. fischeri ES114 reference genomes NC006840, NC006841, and NC006842. Only data from genes with a *P* value of <0.1 and a log ratio value of >2 were considered significant and used for posterior analysis. Using the background libraries described below as control and treatment libraries, we identified an average total of approximately 250 genes for each genotype per strain. These numbers represent 5% of the total genes contained in the V. fischeri genome, including two chromosomes and one naturally occurring plasmid. The validity of our transcriptomic analysis is shown by significant correlations among the members of a set of 15 selected genes amplified with reverse transcriptase quantitative PCR (RT-qPCR) (18). For a more detailed description of the genes, see Table 1 and Fig. S1 in the supplemental material.

**TABLE 1:** Genes related to the initial stages of biofilm formation identified to be upregulated and downregulated in the wrinkly phenotype

Gene and category	Length (bp)	Gene product
Upregulated		
VF_0012	2,418	DNA gyrase subunit B (*gyrB*)
VF_0013	435	Heat shock chaperone (*ibpA*)
VF_0023	981	Oxidoreductase Zn-dependent and NAD binding
VF_0041	1,230	Multidrug efflux system protein
VF_0076	618	Cytochrome *c*_4_
VF_0086	1,185	Oxidoreductase
VF_0099	1,833	GTP binding protein
VF_0114	720	Osmolarity response regulator
VF_0115	1,305	Osmolarity sensor protein
VF_0122	939	Lipid A biosynthesis lauroyl acyltransferase
VF_0145	1,059	Mannose-1-phosphate guanylyltransferase
VF_0146	981	Oxidoreductase
VF_0162	1,152	Exopolysaccharide export protein
VF_0204	291	Cochaperonin GroES
VF_0218	1,932	ABC transporter ATP binding protein
VF_0327	687	ABC transporter ATP binding protein
VF_0356	1,464	MSHA^*a*^ biogenesis protein MshI
VF_0357	639	MSHA biogenesis protein MshJ
VF_0359	1,641	MSHA biogenesis protein MshL
VF_0360	849	MSHA biogenesis protein MshM
VF_0361	1,146	MSHA biogenesis protein MshN
VF_0362	1,725	MSHA biogenesis protein MshE
VF_0363	1,227	MSHA biogenesis protein MshG
VF_0365	564	MSHA pilin protein MshB
VF_0366	465	MSHA pilin protein MshA
VF_0367	597	MSHA pilin protein MshC
VF_0368	582	MshD protein
VF_0369	726	MSHA pilus assembly protein MshO
VF_0370	393	MSHA biogenesis protein MshP
VF_0371	3,108	MshQ protein
VF_0492	879	Collagenase-like protease YhbV
VF_0493	1,002	Collagenase-like protease YhbU
VF_0557	1,668	ABC transporter ATP binding protein
VF_0828	786	Zinc ABC transporter membrane protein
VF_0829	771	Zinc ABC transporter ATP binding protein
VF_0830	894	Zinc ABC transporter periplasmic substrate binding protein
VF_0851	1,692	Acyltransferase
VF_0884	672	ABC transporter ATP binding protein
VF_0885	2,454	ABC transporter permease
VF_0950	522	Holliday junction resolvase
VF_0951	624	Holliday junction DNA helicase RuvA
VF_0952	1,014	Holliday junction DNA helicase RuvB
VF_1194	654	Metal binding protein
VF_1722	912	DNA binding transcriptional activator 2C homocysteine binding
VF_2572	885	Chromosome partitioning protein ParB
VF_2573	798	Chromosome partitioning protein ParA
VF_B0055	1,035	Channel protein VirB6
VF_B0042	1,191	Channel protein VirB10

Downregulated		
VF_0714	765	Flagellar motor protein PomA
VF_0715	930	Flagellar motor protein MotB
VF_0724	315	Regulator of penicillin binding proteins and beta lactamase transcription
VF_0791	882	Transcriptional activator ToxR

a MSHA, mannose-sensitive hemagglutinin.

**FIG 2:**
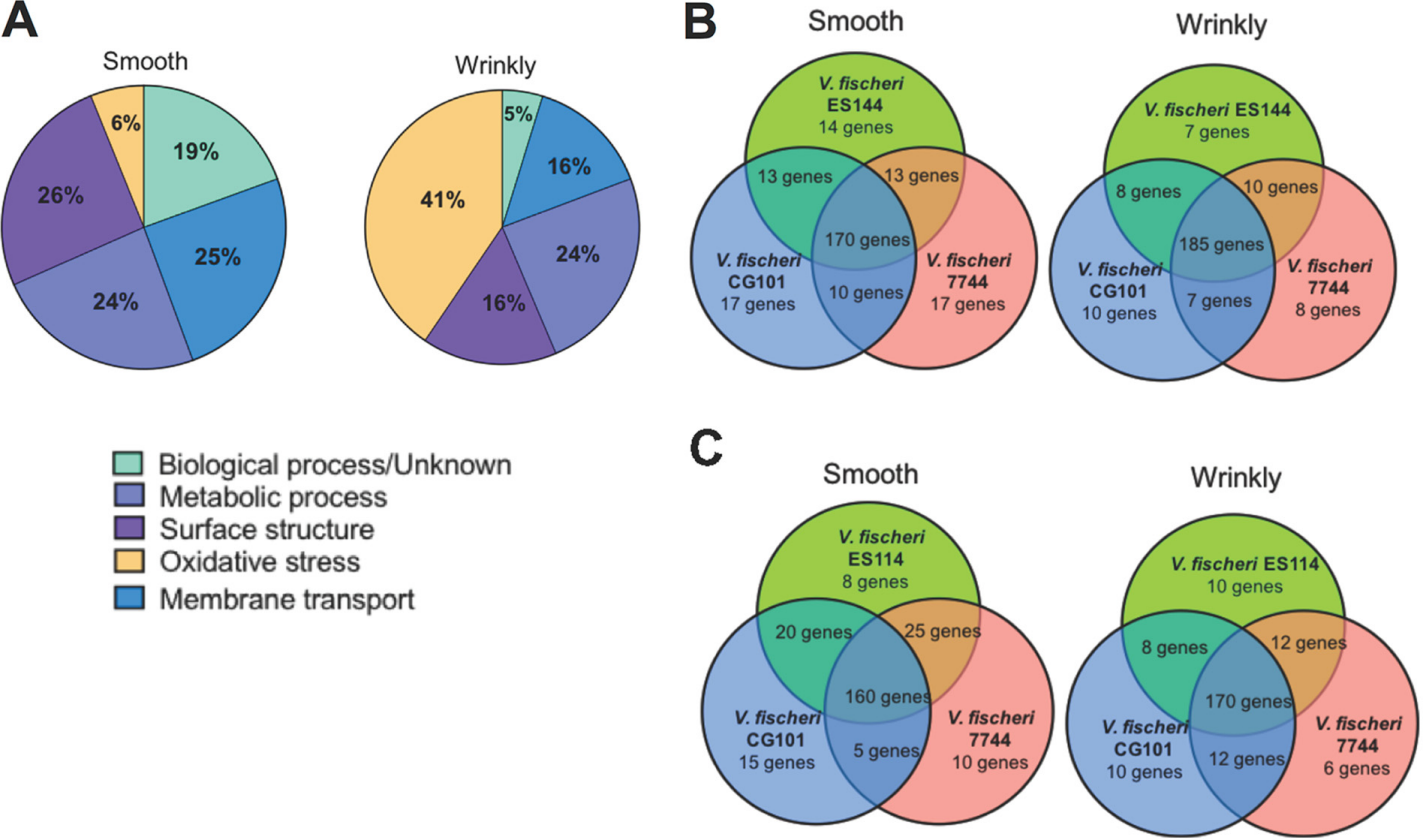
Distribution of genes found in the different phenotypes of Vibrio fischeri. (A) Percentages of genes categorized in the smooth and wrinkly phenotypes. Genes are grouped into 5 main categories (see Table 1 data for a complete list of genes). (B) Upregulated genes are clustered into Venn diagrams indicating the number of genes that strains share (total number of genes found = 210 for each phenotype). (C) Downregulated genes are clustered into Venn diagrams indicating the number of genes that strains share (total number of genes found = 200 for each phenotype).

**FIG 3:**
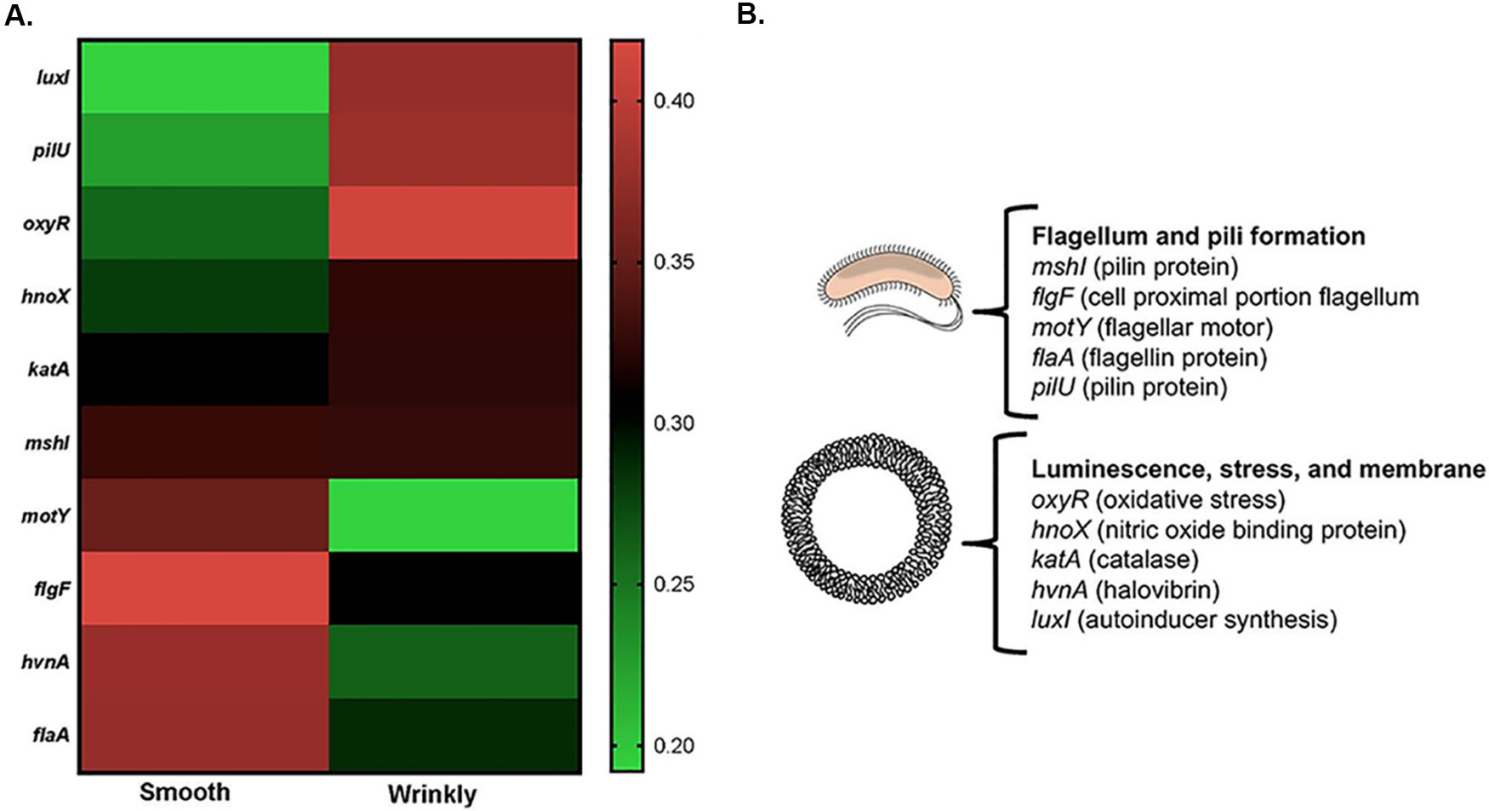
Summary and selection of genes implicated in smooth and wrinkly phenotypes. (A) Heat map indicating genes upregulated and downregulated. Data are shown as a colored map reflecting logarithms related to genetic changes (red areas indicate an increase in gene expression; green areas indicate a decrease in gene expression). (B) Summary of the function of selected genes. Genes are clustered in two main categories: motility processes (flagellum and pilus formation) and biological processes (luminescence, stress, and membrane transport).

**FIG 4:**
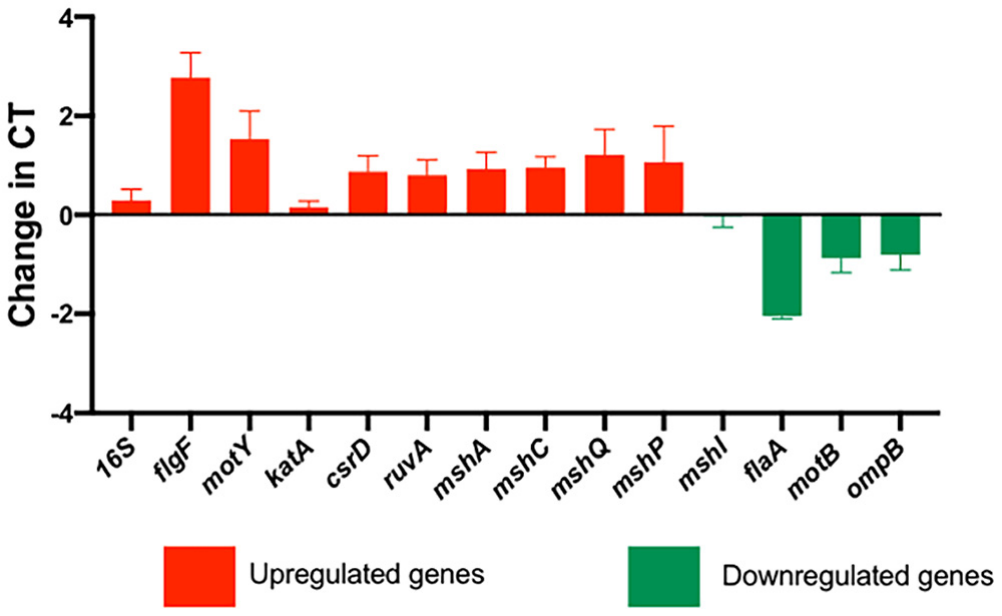
Validation of gene expression by transcriptome analysis. Cross threshold values (CT) of selected genes are represented for an average of 3 biological replicates and 3 technical replicates per phenotype of strain ES114.

The strains selected for our study are from significantly different environments (host environment or free-living environment), and we hypothesized that differential analysis would reveal variations among all three strains. Surprisingly, our analysis demonstrates similar patterns of gene regulation between the strains of either wrinkly or smooth isolates, with only a 2% variation of both upregulated and downregulated clusters of genes per category. However, our report provides new insights into phenotype-specific transcriptomes and reveals that common genes could modulate the transition between the smooth and wrinkly phenotypes despite high conservation at the DNA level (19). Figure 2A presents a summary of the genes detected according to different metabolic and physiological categories, where the Venn diagrams indicate the number of genes that are shared between bacterial clones (Fig. 2B and C). The heat map (Fig. 3) summarizes gene expression of loci selected for RT-PCR analysis (Fig. 4).

### Biosynthetic and metabolic pathways.

The set of genes classified in this category include essential housekeeping genes responsible for cell growth and cell division. Overall, expression levels of the number of genes within this category were not significantly different in the wrinkly and smooth isolates; however, one important difference was noted when a set of genes responsible for biosynthesis of flagella showed a significant decrease in expression within the wrinkly phenotype. The results showed overexpression of the *flaA* and *motY* genes (responsible for C ring synthesis and hook flagellar synthesis components [20, 21]). A significant decrease in expression of *flaA* was observed in the wrinkly phenotype, which is the “master regulator” gene of flagellar hierarchy (20). Previous studies have reported that flagellum-dependent motility is not essential for the wrinkly colony phenotype and that, as a consequence, such colonies also show decreased biofilm formation. Such trait loss in the wrinkly phenotype allows rapid adaptation by compensating with increases in polysaccharide production and biofilm formation (properties discussed below) such as have been previously reported in other bacterial strains with similar wrinkly phenotypes (9).

### Surface structure, membrane transport, and cell-cell communication.

**FIG 5:**
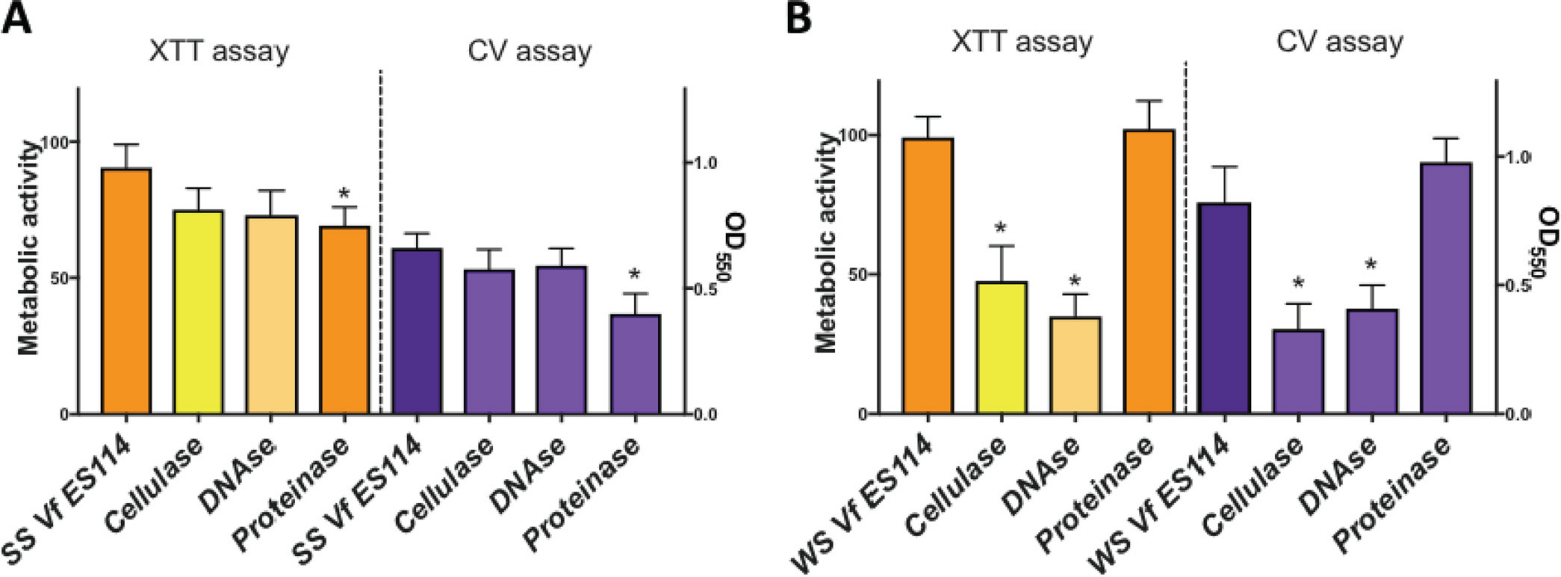
Biofilm formation of smooth spreader (SS) V. fischeri (*Vf*) ES114 (A) and wrinkly spreader (WS) V. fischeri ES114 (B). The following two quantitative biofilm assays were performed: metabolic XTT and crystal violet. Totals of 5 technical replicates and 3 biological assays were performed (*P* < 0.05).

The evolutionary and ecological success of wrinkly spreaders can also be explained by the ability to rapidly establish substantial biofilm growth (7, 16). Proliferative biofilm growth leads to increased survival and better access to oxygen, which permits maximal ATP generation via the electron transport chain. Many mechanisms of cell-cell communication have been found in wrinkly spreaders of Pseudomonas fluorescens (9). Most of these mechanisms are reflected in a set of genes identified as upregulated in the wrinkly phenotype and categorized under cell communication and membrane transport (Fig. 2A). Interestingly, in P. fluorescens, similar genes responsible for the induction of various quorum sensing pathways (such as c-di-GMP metabolism) were also detected (9). c-di-GMP levels influence a wide range of phenotypes and cellular responses that can be differentially regulated in the two phenotypes studied here. For example, there can be clear differences in secretion, motility, synthesis of secondary metabolites, and production of quorum sensing molecules (22). The link of c-di-GMP production and gene expression needs to be studied in more detail to establish a clear role of this second messenger in both wrinkly and smooth phenotypes. One interesting study identified c-di-GMP as a regulator of cellulose synthase activity, which would explain why the biofilm matrix is composed of high cellulose content within the wrinkly spreader (Fig. 5). Our screen also uncovered multiple genes that affect c-di-GMP metabolism. However, expression of multiple unknown regulators made it difficult to establish the correlation of this second messenger with our two different phenotypes (11). Future studies will include a combinational approach of differential expression/transcriptome analyses with site-directed mutational studies of targeted genes that will help elucidate and provide a clearer idea of the role of specific regulons and networks involved in production and function of c-di-GMP.

Quorum sensing (QS) is a phenomenon that allows bacteria to sense small molecules (autoinducers [AI]) whose numbers increase with population density and to activate cascades of gene regulation for different operons (23). AIs are classified into three types based on their synthesis pathways, i.e., autoinducer 1, autoinducer 2, and autoinducer 3, and autoinducing peptides (23). *Vibrio* species mostly produce the first two types of autoinducers, whose common structure is composed of a hydrophilic homoserine lactone ring and a hydrophobic acyl side chain that can be short (<8 carbons) or long (≥8 carbons). Our study demonstrated that there was no clear difference between the expression levels of genes responsible for the short-side and long-side carbon chain autoinducers. However, there are clear differences in expression of the protein transporters responsible for releasing some of these structures into the environment (24). Short-chain autoinducers can move across cell membranes upon synthesis, whereas long-chain autoinducers can be released only through the activity of efflux pathways (23, 24). Our study showed upregulation of genes that synthesize the Omp pump (Fig. 2), which is responsible for active transport of autoinducers in the wrinkly spreader. This leads to an increase in the release of long-chain autoinducers that eventually trigger quorum formation and biofilm synthesis. Another interesting gene that was found to be differentially expressed is *ompU*, which was upregulated by >2-fold in the wrinkly phenotype. The OmpU protein is part of a well-characterized group of outer membrane proteins that act as adhesins and that have been reported to be linked to virulence regulators and, in the case of V. fischeri, to play a role in colonization of squid hosts. Additionally, previous studies in our laboratory showed an increase in OmpU protein quantities in biofilms formed by the symbiotic V. fischeri ES114 strain (25, 26). In summary, upregulation of QS protein transporters and membrane adhesins seems also to play a role in the transition from the smooth to the wrinkly phenotype.

One of the most fascinating properties of V. fischeri is its ability to produce light during *in vitro* increased bacterial growth and, most importantly, during the infection of its squid or fish hosts, allowing establishment of well-known mutualisms (27). Bioluminescence is regulated by a coordinated activation of luminescence or *lux* genes, orchestrated by transcriptional regulators also involved in quorum sensing responses (28). *luxAB* genes encode the luciferase enzyme (essential for luminescence), *luxCDE* genes synthesize the aldehyde substrate, and both groups of genes (contained in a single operon) are regulated by *luxR* and *luxI*, which are also responsible for quorum sensing production. Notably, our study revealed differences in the levels of expression of *luxA*, which were increased in the wrinkly spreaders, possibly explaining the increased infection fitness of these colonies (Fig. 3 to 5) (19). Additionally, *luxI* was downregulated in the smooth phenotype, which might provide a partial explanation of the decrease in biofilm formation of this phenotype (linked to QS production). However, other QS-related genes were not affected overall, indicating that *luxI* plays an unknown role in smooth colonies that remains unrevealed.

### Stress-related genes.

V. fischeri is a highly adaptable organism, a quality that enables it to overcome the effects of changing hostile environments, including nutrient limitation, exposure to reactive forms of oxygen, protozoan predation, and temperature and pH fluctuations (29–32). One of the major stressors that V. fischeri must overcome is exposure to toxic radical species that are abundant in marine systems, including reactive oxygen species (ROS). Wrinkly spreader phenotypes may experience increased exposure to ROS, given the morphology of the pellicle and the higher surface area exposed to oxygen (5). Additionally, V. fischeri encounters oxidative stress during host infection in the form of peroxide and superoxide radicals. Catalases also represent crucial components for ROS survival and resistance (33). In V. fischeri, catalase activity is localized in the periplasm of wild-type V. fischeri cells and has been identified as being associated with KatA (33). KatA has been previously described and was reported to be crucial for detoxification of hydrogen peroxide coming from the external environment and was also described to be important for host colonization and biofilm formation (33). Interestingly, the *katA* gene, as well as other major transcriptional regulators of oxidative stress, was found to be upregulated in the wrinkly morphology in wrinkly V. fischeri (Fig. 3 and 4). These findings highlight the correlation of the wrinkly phenotype with stress survival and host colonization in V. fischeri.

Molecular-chaperone-related genes were also found to be upregulated in the wrinkly phenotype. Most chaperones are stress-related factors that are important in assisting with protein folding and with refolding of denatured structures (34). Genes responsible for major chaperone synthesis (such as *dnaK*) were found to be upregulated in wrinkly colonies, potentially assisting in survival under extremely stressful conditions. More importantly, of particular interest in this study was the upregulation of the *hfqA* gene, which is a chaperone that binds to small RNAs (sRNAs) to regulate transcription of luminescence and surface adherence, properties associated with the molecular characteristics of the wrinkly phenotype. Previous studies have shown that such chaperones (such as DnaK) are involved in the formation of Escherichia coli biofilms during cellular stress (35).

**FIG 6:**
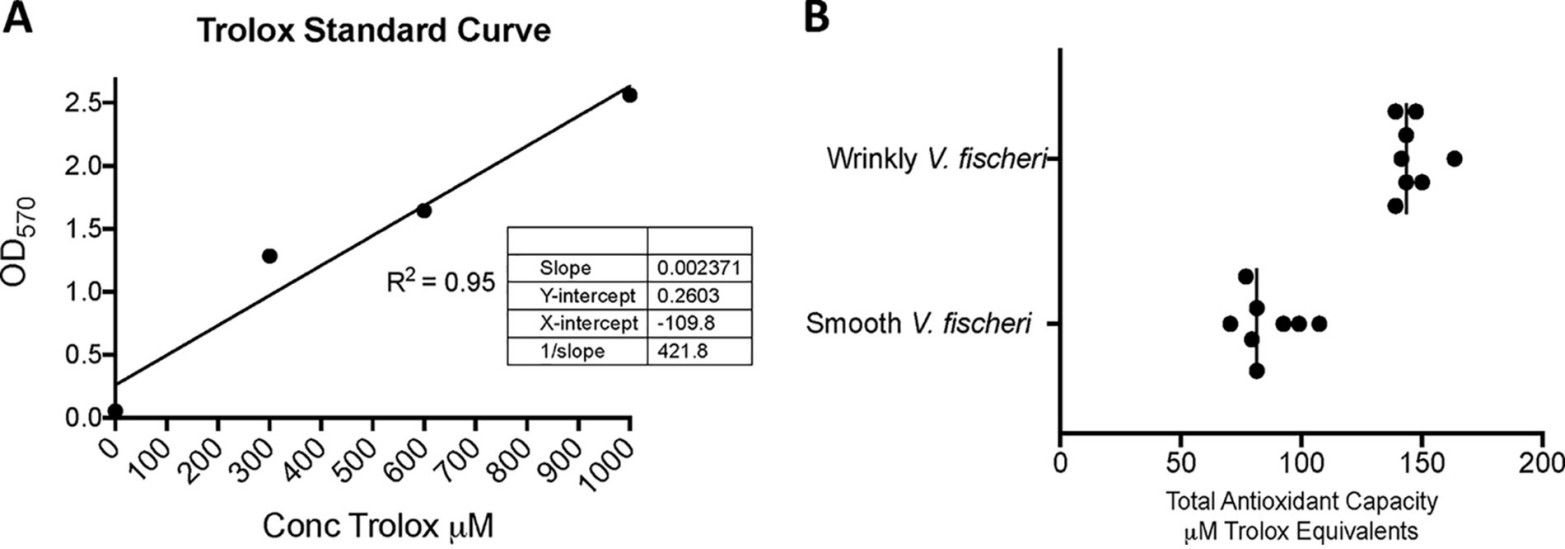
Calculation of antioxidant capacity in smooth and wrinkly V. fischeri strains. (A) Standard curve calculated in micromolar units based on the capacity with which cupric ions (Cu^2+^) are reduced by antioxidants to cuprous ions (Cu^+^); the resulting ions formed a colored complex proportional to the level of TAC in the sample. (B) The total antioxidant capacity of strains was determined by calculating the optical density of cell preparations (*n* = 8) divided by the standard curve slope. There was a significant difference in antioxidant capacity between strains that indicated an increase in TAC in the wrinkly variant.

We performed a test that detects total antioxidant capacity (TAC) to further evaluate the differences in antioxidant production between the wrinkly and smooth phenotypes of V. fischeri. Our results indicate that there was a significant difference between the smooth and wrinkly phenotypes in terms of antioxidant production (Fig. 6). This assay further validated our results with regard to upregulation of antioxidant-related genes. The total antioxidant capacity assay is based on reduction of copper(II) to copper(I), allowing measurement of the differences using a chromogen and of optical density at 570 nm (OD_570_).

### Assessment of biofilm formation and effect of degrading enzymes.

The mannose-sensitive hemagglutinin cluster of genes was previously reported to be important for host colonization and the initial stages of biofilm formation (particularly the attachment stage) in different species of *Vibrio*, including V. cholerae and V. fischeri (36, 54). *msh* genes were found to be upregulated in the wrinkly phenotype, which has been observed to form stronger biofilms than the smooth counterpart. Similarly, genes related to polysaccharide biosynthesis have been identified to be upregulated in the wrinkly phenotype, including the sensor kinase regulator gene *rcsS*, which is responsible for activating the symbiosis polysaccharide locus (*syp*) important for host colonization and biofilm formation (37). Future studies will include a more robust analysis of gene expression of the *syp* cluster and its regulators in smooth and wrinkly colonies.

The composition of V. fischeri biofilms has been studied in the past by utilizing different quantitative and qualitative methods. In this study, we selected two methods to measure the amount of biofilm formed by the wrinkly and smooth morphotypes and the accompanying effect that hydrolyzing enzymes had on the biofilms. Results obtained from the biofilm assay (Fig. 5) are quite intriguing. Our data confirm that both phenotypes of V. fischeri can produce biofilms, with the extracellular matrix comprised of nucleic acids, proteins, and polysaccharides, including cellulose. Cellulose, protein, and DNA have previously been shown to be building materials in *Vibrio* biofilms (38–40). Interestingly, within the V. fischeri wrinkly spreader phenotype, mutations were identified in loci that are associated with Holliday junctions in nucleic acid (Table 1). For extracellular DNA that contributes to the extracellular matrix, Holliday junctions are important for maintaining biofilm integrity and stability in bacteria (41). Holliday junctions are analogous to “reinforced concrete,” since the hydrogen bonding between nucleotides fortifies the extracellular matrix of biofilms. Holliday junctions can be destabilized by nucleases (42), which may explain why DNase diminished biofilm formation in the V. fischeri wrinkly spreader phenotype. Clearly, our data from the biofilm assays (Fig. 5) demonstrate that bacterial biofilms can be constructed in alternative ways. For instance, in this study, wrinkly spreader biofilms appear to possess more cellulose and DNA than biofilms corresponding to the smooth phenotype.

Polysaccharide is usually the predominant building material in the biofilm extracellular matrix compared to other macromolecules. However, there have been reports of instances where the biofilm extracellular substance consisted chiefly of protein, including S-layers (43, 44). For example, extracellular protein in Pseudomonas putida biofilms constitutes 75% of the material present in the water-soluble fraction (45, 46). Moreover, *V. salmonicida* is believed to be capable of producing biofilms with a substantial amount of “S-layer” type proteins, which occur and are shed extracellularly in this species (8). Unfortunately, the upper limit of total protein content in V. fischeri biofilms is still unknown. Future research will include identifying other components that can serve as building materials for V. fischeri biofilms, including dextrin, fucans, and alginate (9). Interestingly, the biofilm matrix of the smooth phenotype showed a significant decrease with proteinase K. Forthcoming research will include examining the protein and lipid content present in V. fischeri biofilms. For instance, future work should include biofilm assays that utilize lipases and lectinases to determine the extent to which V. fischeri employs such proteins as well as glycerides and wax esters to construct material for the extracellular biofilm matrices.

### Perspectives and outlook.

Novel mutations were responsible for these evolutionary changes. Here, we highlighted the expression patterns of some of the most fascinating genes related to the wrinkly spreader phenotype (Fig. 1 and 2) and their functions related to survival, adaptation, and squid host colonization (Fig. 3 and 5). We had also previously observed that stress-related genes are important for host colonization and repair mechanisms; however, in the present study we included only the genes that were previously reported to be differentially regulated during host colonization and biofilm formation, important features for symbiotic competency. Our study was also validated by the analysis of 15 corresponding genes using quantitative reverse transcriptase PCR (qRT-PCR) (Fig. 4) (18). Thus, the present work demonstrates differences induced by positive selection pressure for increased biofilm formation, which is the driver for microbial adaptive radiation and the generation of the wrinkly colony morphology (5). Note that this analysis is preliminary and opens the way to more-specialized and functionary studies for each of the strains analyzed in this study.

Given how little we understand gene expression in bacteria that employ different life history strategies daily, this report provides some preliminary insight into the genetic fingerprint behind regulation of biofilm formation and represents a pioneer analysis of molecular insights into two major phenotypes observed in V. fischeri. The discovery of multiple genes involved in the regulation of transitions between the two different phenotypes described here not only has provided important insights into the forces driving the biphasic life history strategy of this beneficial symbiosis but also warrants questions about the circuitry governing colony phenotypes. We realize that our transcriptome screen represents just an initial survey of a more complex set of regulatory pathways, and we anticipate that more genes, proteins, metabolites, and molecular markers remain to be discovered and linked to the transition between wrinkly and smooth phenotypes.

## MATERIALS AND METHODS

### Bacterial growth conditions.

Smooth and wrinkly colonies were grown in triplicate from single colonies displaying the particular phenotype. The strains selected include the free-living isolate V. fischeri ATCC 7744 and the symbiotic isolates ES114 and CG101. Strains were initially streaked onto modified seawater-tryptone (MSWT) (5)–agar (2.0%) and grown overnight at 28°C, with single colonies chosen and subcultured into MSWT liquid medium and grown to an OD_600_ of 0.3 at 28°C. The growth dynamics of the cultures was followed by measuring optical density at 600 nm (considering that an OD_600_ of 1 equals 1 × 10^8^ CFU/ml). Cultures (500 μl) were transferred into 600 μl of RNAprotect solution (Qiagen, Valencia, CA) for RNA storage as previously described (18).

### RNA extraction and sequencing.

Total RNA was extracted from the stored RNAprotect solution using an RNAeasy Qiagen kit (Qiagen, Valencia, CA) as indicated by the manufacturer’s instructions, followed by an additional treatment with DNase I (Qiagen, Valencia, CA) to remove traces of DNA. rRNA depletion and mRNA enrichment were performed using an Illumina Ribo-Zero rRNA removal kit (catalog no. MRZ116C) according to the instructions of the manufacturer (Illumina, San Diego, CA). RNA concentrations were measured using an RNA Qubit assay (Invitrogen, Burlington, Ontario, Canada), and RNA integrity was evaluated using a Bioanalyzer RNA Pico assay (Agilent Technologies, Santa Clara, CA) (18). cDNA libraries were constructed using a ScriptSeq Complete kit for bacteria (Epicentre, Madison, WI). cDNA was then purified using an Agencourt AMPure XP system (Beckman Coulter, Beverly, MA), and the second cDNA was generated by adding the Illumina adapters as the forward primer and a ScriptSeq index primer as the reverse primer. The resulting libraries were purified using an AMPure XP system (Beckman Coulter, Beverly, MA), quantified with the DNA Qubit assay, and evaluated using the Bioanalyzer sensitivity DNA assay (Agilent Technologies, Santa Clara, CA). One hundred single reads were generated using the Illumina HiSeq 2000 platform at the National Center for Genome Research in Santa Fe, NM.

### Bioinformatic analysis.

The reads were mapped against the V. fischeri ES114 genome (taxonomy identifier [ID] 312309) using EDGE-pro v1.3.1. Only genes with a fold change value higher than 2 and a false-discovery-rate (FDR) value lower than 0.1 were considered significant for this study. The significantly upregulated and downregulated genes were analyzed for gene ontology (GO) term enrichment (*P* value of <0.05), using GOToolBox (http://genome.crg.es/GOToolBox/), and the significantly enriched terms were further explored using the REVIGO Web application to identify and visualize relationships among the GO terms (18, 47).

### Effect of enzymes on biofilm formation.

The effects of three enzymes on biofilms formed by the wrinkly and smooth strains of V. fischeri ES114 were measured. Cultures were grown overnight at 28°C and 250 rpm in MSWT, and biofilm formation assays and quantifications were performed using the following two previously described methods: crystal violet (CV) staining (48) and 2,3-bis-(2-methoxy-4-nitro-5-sulfophenyl)-2H-tetrazolium-5-carboxanilide salt (XTT) assay (49, 50). In brief, overnight isolates were subcultured and grown to a cell density of 1 × 10^9^ CFU/ml. Aliquots of 300 μl were added to individual wells on a Corning flat-bottom polystyrene 96-well microtiter plate (catalog no. CLS3628; Sigma-Aldrich, St. Louis, MO) and incubated for 24 h at 28°C under static conditions. After incubation, non-biofilm formers (planktonic cells) were removed gently using a multichannel pipette and wells were washed 3 times with sterile MSWT medium. The effect of inhibitory enzymes was tested as previously described (51, 52). Volumes of 300 μl of MSWT containing the enzymes cellulase, DNase, and proteinase were added to 5 wells per enzyme, and volumes containing another 300 μl (with no enzyme) were added to 5 wells for the nontreatment control. For negative controls, 5 uninoculated wells were used at the same time. The final concentrations of enzymes were as follows: 20 mg/ml cellulase (catalog no. C1184; Sigma-Aldrich, St. Louis, MO), 0.1% (vol/vol) DNase I (Thermo Scientific, Waltham, MA), and 1% (vol/vol) proteinase K (Qiagen, Hilden, Germany). MSWT preparations with enzymes and without enzymes were incubated with the preformed biofilms for 1h at 28°C under static conditions. After the incubation time, solutions were removed, wells were washed with sterile medium, and CV and XTT assays were performed to quantitatively measure biofilm formation. For the CV assay, 300 μl of an aqueous solution of crystal violet (2%) was added and the resulting reaction mixture was incubated at room temperature for 30 min. CV was then removed, and the plate was washed 10 times with saline solution. CV that had attached to the biofilm cells was then solubilized by adding 95% ethanol, and optical density (*A*_550_) was recorded for each biofilm in the individual wells. For the metabolic XTT reduction assay, 0.010 mol/liter menadione (Sigma-Aldrich, St. Louis, MO) stock acetone solution was mixed with XTT-Ringer’s lactate solution (0.5 g of XTT [Sigma-Aldrich, St. Louis, MO]) and diluted in 1 liter of 1× phosphate-buffered saline (PBS) or Ringer’s lactate solution at a final concentration of 1 μmol/liter. A 300-μl aliquot of the XTT-Ringer’s-menadione solution was then added to each of the wells. Plates were incubated for 2 h at 28°C and covered in aluminum foil (to avoid light degradation), optical density (*A*_490_) was measured after the incubation time, and total metabolic activity was calculated and is indicated as percentages compared to the nontreated control. Reduction of XTT by metabolically active cells resulted in transformation of the original clear/yellow solution to an intense orange solution.

### Total antioxidant capacity determination.

Total antioxidant capacity (TAC) of smooth and wrinkly phenotypes was measured using a colorimetric assay (BioAssay Systems, Hayward, CA). In brief, cells were grown overnight, optical density was measured, and the culture was adjusted to an OD_600_ of 0.5, and a 100-ml volume of cells was washed 3 times with cold PBS solution, subjected to vortex mixing with beads for 10 min, and spun down for 10 min at 4°C. Cell-free supernatants were used for subsequent TAC assay, which was performed according to the manufacturer’s instructions. Results were calculated by comparison with the standard curve, and the total antioxidant concentration was calculated in micromoles (53).

### RT-qPCR.

RT-qPCR was used for validation of gene expression. cDNA was synthesized using iScript reverse transcription Supermix for RT-qPCR (Bio-Rad, Hercules, CA); rRNA-depleted samples as a template; and a reaction incubation program of priming at 25°C for 5 min, reverse transcription at 46°C for 20 min, and RT inactivation at 95°C for 1 min. The qPCRs (10 μl) were performed in triplicate using SsoAdvanced Universal SYBR green Supermix (Bio-Rad, Hercules, CA), and a 0.5 μM concentration of each primer was added along with 1 ng of cDNA as the template. The housekeeping gene selected was the glyceraldehyde phosphate dehydrogenase A gene (*gapA*) (forward primer, AGCTCGTTCAATGCAAGCG; reverse primer, AAACCTTCACGACCCGTGTT). The PCR program was as follows: a polymerase activation and DNA denaturation step of 30 s at 98°C, followed by 40 cycles of denaturation at 98°C for 15 s and primer annealing/extension at 60°C for 30 s. After amplification, a melting curve analysis consisting of 60 cycles of temperature increases from 65 to 95°C at a rate of 0.5°C per cycle was included to assess the specificity of the amplification (18). Three biological replicates were performed. The relative expression levels of the target genes were calculated using *gapA* as the reference gene for each comparison (47).

## Supplementary Material

Supplemental file 1
