# Supplementary material for: Identification of a Transcriptomic Network Underlying the Wrinkly and Smooth Phenotypes of Vibrio fischeri
Source: J Bacteriol. 2021 Jan 11;203(3):e00259-20. doi: 10.1128/JB.00259-20 (PMC7811199; doi:10.1128/JB.00259-20)
Supplement: Supplemental file 1 [file JB.00259-20-s0001.pdf]

Supplementary Figures and Tables

Figure S1. Heat map indicating genes upregulated and downregulated in the wrinkly phenotype of *V. fischeri* ES114, data is shown as a colored map reflecting logarithms that related to genetic changes (red areas indicate an increase in gene expression, green areas depict a decrease in gene expression).

Table S1. List of selected upregulated and downregulated genes from the wrinkly spreader *V. fischeri* ES114.

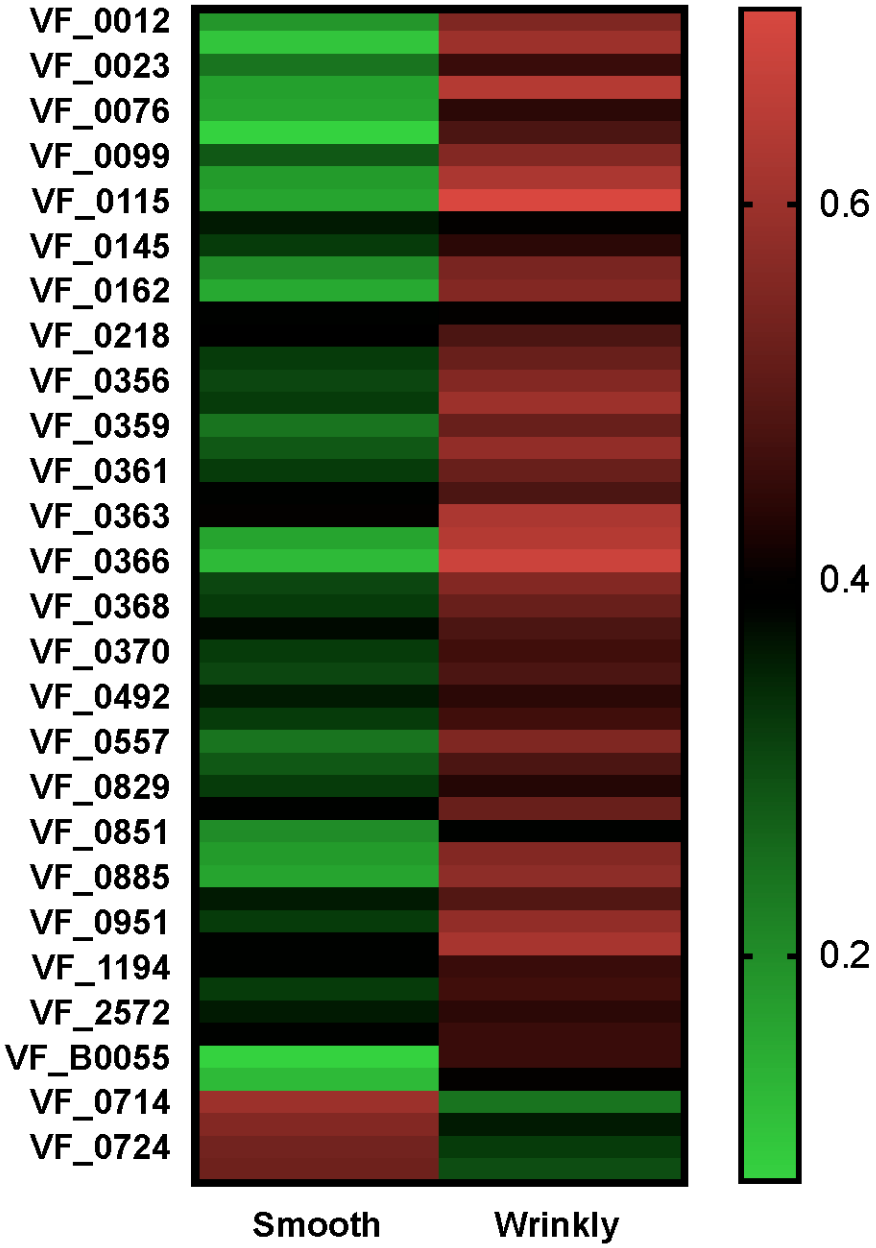
VF_0012
VF_0023
VF_0076
VF_0099
VF_0115
VF_0145
VF_0162
VF_0218
VF_0356
VF_0359
VF_0361
VF_0363
VF_0366
VF_0368
VF_0370
VF_0492
VF_0557
VF_0829
VF_0851
VF_0885
VF_0951
VF_1194
VF_2572
VF_B0055
VF_0714
VF_0724
Smooth
Wrinkly
0.6
0.4
0.2

Table S1. List of selected upregulated and downregulated genes from the wrinkly spreader *V. fischeri* ES114.

VF NUMBER	BP LENGTH	GENE DESCRIPTION
UPREGULATED GENES		
VF_0012	2418	DNA gyrase subunit B (*gyrB*)
VF_0013	435	heat shock chaperone (*ibpA*)
VF_0023	981	oxidoreductase Zn-dependent and NAD-binding
VF_0041	1230	multidrug efflux system protein
VF_0076	618	cytochrome c4
VF_0086	1185	oxidoreductase
VF_0099	1833	GTP-binding protein
VF_0114	720	osmolarity response regulator
VF_0115	1305	osmolarity sensor protein
VF_0122	939	lipid A biosynthesis lauroyl acyltransferase
VF_0145	1059	mannose-1-phosphate guanylyltransferase
VF_0146	981	oxidoreductase
VF_0162	1152	exopolysaccharide export protein
VF_0204	291	co-chaperonin GroES
VF_0218	1932	ABC transporter ATP-binding protein
VF_0327	687	ABC transporter ATP-binding protein
VF_0356	1464	MSHA biogenesis protein MshI
VF_0357	639	MSHA biogenesis protein MshJ
VF_0359	1641	MSHA biogenesis protein MshL
VF_0360	849	MSHA biogenesis protein MshM
VF_0361	1146	MSHA biogenesis protein MshN
VF_0362	1725	MSHA biogenesis protein MshE
VF_0363	1227	MSHA biogenesis protein MshG
VF_0365	564	MSHA pilin protein MshB
VF_0366	465	MSHA pilin protein MshA

VF_0367	597	MSHA pilin protein MshC
VF_0368	582	MshD protein
VF_0369	726	MSHA pilus assembly protein MshO
VF_0370	393	MSHA biogenesis protein MshP
VF_0371	3108	MshQ protein
VF_0492	879	collagenase-like protease YhbV
VF_0493	1002	collagenase-like protease YhbU
VF_0557	1668	ABC transporter ATP-binding protein
VF_0828	786	zinc ABC transporter membrane protein
VF_0829	771	zinc ABC transporter ATP-binding protein
VF_0830	894	zinc ABC transporter periplasmic substrate-binding protein
VF_0851	1692	acyltransferase
VF_0884	672	ABC transporter ATP-binding protein
VF_0885	2454	ABC transporter permease
VF_0950	522	Holliday junction resolvase
VF_0951	624	Holliday junction DNA helicase RuvA
VF_0952	1014	Holliday junction DNA helicase RuvB
VF_1194	654	metal-binding protein
VF_1722	912	DNA-binding transcriptional activator%2C homocysteine-binding
VF_2572	885	chromosome partitioning protein ParB
VF_2573	798	chromosome partitioning protein ParA
VF_B0055	1035	channel protein VirB6
VF_B0042	1191	channel protein VirB10
DOWNREGULATED GENES		
VF_0714	765	flagellar motor protein PomA
VF_0715	930	flagellar motor protein MotB
VF_0724	315	regulator of penicillin binding proteins and beta lactamase transcription
VF_0791	882	transcriptional activator ToxR
